# Autoantibodies and the Immune Hypothesis in Psychotic Brain Diseases: Challenges and Perspectives

**DOI:** 10.1155/2013/257184

**Published:** 2013-08-24

**Authors:** Karrnan Pathmanandavel, Jean Starling, Russell C. Dale, Fabienne Brilot

**Affiliations:** ^1^Neuroimmunology Group, Institute for Neuroscience and Muscle Research, The Kids Research Institute at the Children's Hospital at Westmead, University of Sydney, Locked Bag 4001, Westmead, NSW 2145, Australia; ^2^Discipline of Paediatrics and Child Health, Sydney Medical School, University of Sydney, Westmead, NSW 2145, Australia; ^3^The Walker Unit, Concord Centre for Mental Health, Concord West, NSW 2138, Australia; ^4^Discipline of Psychiatry, Sydney Medical School, University of Sydney, Sydney, NSW 2006, Australia

## Abstract

The pathophysiology of psychosis is poorly understood, with both the cognitive and cellular changes of the disease process remaining mysterious. There is a growing body of evidence that points to dysfunction of the immune system in a subgroup of patients with psychosis. Recently, autoantibodies directed against neuronal cell surface targets have been identified in a range of syndromes that feature psychosis. Of interest is the detection of autoantibodies in patients whose presentations are purely psychiatric, such as those suffering from schizophrenia. Autoantibodies have been identified in a minority of patients, suggesting that antibody-associated mechanisms of psychiatric disease likely only account for a subgroup of cases. Recent work has been based on the application of cell-based assays—a paradigm whose strength lies in the expression of putative antigens in their natural conformation on the surface of live cells. The responsiveness of some of these newly described clinical syndromes to immune therapy supports the hypothesis that antibody-associated mechanisms play a role in the pathogenesis of psychotic disease. However, further investigation is required to establish the scope and significance of antibody pathology in psychosis. The identification of a subgroup of patients with antibody-mediated disease would promise more effective approaches to the treatment of these high-morbidity conditions.

## 1. Introduction 

Psychotic disorders are severe mental illnesses where thoughts or behaviours are out of touch with reality. Common symptoms include hallucinations (perceptions in the absence of a stimulus), delusions (fixed false beliefs), and irrational behavior [1]. The two highest prevalence psychotic disorders are schizophrenia and bipolar affective disorder, but generally psychotic disorders are heterogeneous and their causes poorly understood. Patients with schizophrenia have multiple medical comorbidities [2] and a ten-year reduction in life expectancy [3]. The financial, social, and human costs of psychotic disorders are high [4]. 

A number of hypotheses to explain the aetiology of psychosis exist, and it is likely that the disease process is multifactorial and complex. For the last few decades the prevailing model for the pathogenesis of psychotic illness has been neurodegenerative, with initial studies demonstrating broad pathological changes in brain parenchyma, such as ventricular enlargement, decreased gray and white matter volumes, decreased overall brain volume [5], and cognitive decline [6]. This model focuses on the dopaminergic system [7, 8], and it is noteworthy that all clinically effective antipsychotics have some degree of dopamine-blocking capability [9]. More recently the focus has shifted to a “disconnection syndrome” [10], where incoming neural activity is poorly integrated across wide regions of the brain. Drugs such as phencyclidine and ketamine, both N-methyl-D-aspartate (NMDA) glutamate receptor antagonists, mimic this syndrome and produce similar syndromes to schizophrenia [11, 12]. However, both theories have the dysfunction of neuronal receptor populations in common, whether localised or diffuse, transient or enduring. 

The role of the immune system in the development of psychotic psychiatric diseases has been extensively investigated [13]. In particular, some studies suggest that an inflammatory autoimmune process may be of pathogenic significance in a subgroup of psychotic patients [14, 15]. While the immune hypothesis has been gaining momentum for some time, recent developments in the study of antibodies associated with central nervous system (CNS) autoantigens have suggested a promising new focus for future investigations. In particular, the detection of autoantibodies against neuronal receptors [16–18] suggests a link between the receptor dysfunction paradigm in psychosis and an as yet unclear spectrum of immunological abnormalities. 

## 2. The Immune Hypothesis in Psychosis 

The immune hypothesis in psychosis has been evolving for decades. Abnormal activation of the immune system may be a feature of psychotic disease, in particular schizophrenia [14, 15]. Immune hypotheses were motivated initially by the clinical and epidemiological features of the disease [14, 19] and then by accumulating serological evidence of changes in immune system function [20, 21]. 

Historically, it is likely that a link was made between the immune system and psychosis because of the perception that infection had a causative role [22]. Interest in the epidemiology of psychotic disease has been nourished from 1845 [23] to the present [24] by the description of correlations between psychosis and infectious agents, pandemics, and autoimmune disease. Recent epidemiological studies suggest that there is a subgroup of patients who may have a shared predisposition to both immune and psychiatric disease [25, 26]. 

Although initially grounded in circumstantial evidence, the immune hypothesis of psychosis now encompasses many related fields of investigation. Studies of serum samples have demonstrated elevated levels of some cytokines, morphologically abnormal lymphocytes, and elevated C-reactive protein, a nonspecific marker of inflammation [27, 28]. Elevated immune-related gene expression has also been observed in brain tissue [29]. Imaging studies have highlighted the abnormal quantity and localisation of microglial populations [27]. The paradigm of autoantibody-associated neuropsychiatric disease has found application in schizophrenia, with the detection of anti-neurotransmitter receptor antibodies in a subgroup of patients [16–18]. As is noted below, the progress of research to substantiate the immune hypothesis has demonstrated a trend towards greater consistency and specificity of results. 

## 3. Immunological Abnormalities in Psychosis 

A wide range of immune effectors has been measured in patients who suffer from psychosis [13, 20, 36]. Experimental work has made use of peripheral blood samples, cerebrospinal fluid (CSF) samples, biopsies of brain, and a number of *in vitro *and animal models. A crucial link between immunopathological findings in the CNS and the periphery is the ability of immune effectors to traverse the blood brain barrier. The role of the blood brain barrier in influencing the passage of immune modulators has been reviewed extensively elsewhere [37, 38]. The view of the blood brain barrier as a substantially impermeable obstacle to agents of the immune system has been replaced by a model predicated on a more subtle, dynamic regulatory process, in which a low level of lymphocytes survey the brain parenchyma in an essentially random manner. Once exposed to relevant stimulus, the lymphocytes are capable of clonal expansion and executing effector functions. While the passage of cytokines across the blood brain barrier is not substantially impeded and is in fact crucial in mediating the interaction between the immune system and the CNS, the levels of peripheral lymphocytes in brain tissue are normally low. Dysfunction of the blood brain barrier has been detected in patients with psychosis [39] and may play a role in perpetuating and exacerbating pathological immune system activation. Disruption of the blood brain barrier may not, however, be a necessity for immune-mediated mechanisms to influence the brain parenchyma. It is generally agreed that activation of inflammatory agents increases the permeability of the blood brain barrier to immune effector cells [40, 41], potentially laying the foundation for immune-mediated psychopathology. 

Immune system dysfunction in psychosis may arise, in part, from a genetic predisposition. Variants of immune system genes have been associated with schizophrenia, as demonstrated by a recent meta-analysis of genome-wide association studies [42]. It should be noted, however, that not all identified variants have been specifically associated with schizophrenia [43, 44], raising the possibility that immune activation may play a role in a broader range of psychiatric disease. There may be common genetic risk factors that give rise to psychotic disease and established atopic and autoimmune syndromes [25, 26, 45, 46]. Immune dysfunction could define a shared vulnerability to psychiatric disease, which is likely fully manifested in only a subgroup of individuals. 

Many groups have examined the role of cytokines in psychosis (reviewed in [47, 48]). However, it is only recently that a suitably nuanced view of cytokine alterations has emerged, following meta-analysis of a large number of inconsistent studies [49]. Miller and colleagues reported that two categories of cytokines emerged from meta-analysis [49]. IL-1*β*, IL-6, and TGF-*β* were identified as state markers—cytokines that were elevated in acute relapses of disease and in the acute first episode of psychosis. IL-12, IFN-*γ*, TNF-*α*, and sIL-2R (soluble IL-2 receptor) were identified as trait markers and as such were generally elevated, independent of psychotic episodes, or antipsychotic treatment. The role of these cytokines in the formation or maintenance of the disease state remains unclear [50], but the prevalence of these changes in patients with psychosis may be quite high. For example, Fillman and colleagues recently reported inflammatory pathway changes in approximately 40% of a cohort of schizophrenic patients [29]. In the CNS, the modulatory effects of cytokines are thought to be important in dopaminergic, serotonergic, noradrenergic, and cholinergic neurotransmission [36]. Therefore, one hypothesis holds that cytokine-mediated events are the key pathogenic event in schizophrenia [51]. Alternatively, cytokines may be expressed as part of a broader immune response, and their main usefulness in a clinical setting could be as biomarkers. 

Changes in the number, function, and morphology of immune cells have also been reported in psychosis. Recently, García-Bueno and colleagues reported a significant increase in some components of the intracellular NF*κ*B-triggered proinflammatory pathway in peripheral mononuclear blood cells, coincident with a decrease in anti-inflammatory pathways [52]. Both increased numbers and abnormal localisation of microglia in the CNS have also been reported [29, 53], findings that are consistent with the hypothesis that aberrant microglial activation plays a pathogenic role in schizophrenia (reviewed in [54]). Analogous approaches to lymphocytes in peripheral blood, despite being the subject of sustained research interest, have yielded highly heterogeneous results (reviewed in [20]), making it difficult to infer the pathogenicity of various cell lines from abnormal cell counts. 

The immune hypothesis in psychosis is further supported by the observation that anti-inflammatory therapy improves clinical outcome in patients with schizophrenia [55]. A meta-analysis examining the use of nonsteroidal anti-inflammatory drugs (NSAIDs) in schizophrenia found that augmentation of antipsychotics with NSAIDs reduced symptom severity [56]. Antipsychotic drugs have been reported to have immunomodulatory effects of their own that are still not completely understood [57–59]. This phenomenon suggests that some of the symptom relief provided by antipsychotics may be a consequence of immune modulation, but also that studies of the immune system are likely confounded by the effects of antipsychotic medication on the immune system of psychotic patients. 

Patients with psychiatric disease frequently suffer from a range of medical comorbidities [2]. While the most common of these are cardiovascular, malignancies, and respiratory diseases, often related to smoking or the metabolic syndrome induced by psychotropic drugs [60, 61], there is also an increase in infectious or inflammatory pathology in this patient group [14, 15]. The effect of comorbidities in altering immune system function is a significant confounding factor in the analysis of the immune system parameters of patients with psychosis. 

A related hypothesis proposes that prenatal exposure to infectious and inflammatory pathology causes neurodevelopmental injury, potentially manifesting as psychosis [62]. The role of maternal infection as a causative factor in infant pathology has been well established in a range of nonpsychiatric diseases [63]. In pregnancy, a maternal inflammatory state has been associated with an upregulation of inflammatory markers, such as cytokines, which seem to be capable of crossing the placenta and acting upon the foetus [64]. Furthermore, prenatal exposure to infectious and inflammatory pathology has been reported to be a risk factor for adult schizophrenia in a systematic review of population studies [65]. Thus, neurodevelopmental abnormalities following maternal inflammation may account for some of the observed genetic predisposition in schizophrenia. This hypothesis has been reviewed thoroughly by Miller and colleagues [62]. 

## 4. Autoantibodies in Psychosis 

In the last decade, a number of studies have detected antibodies against neuroreceptors or synaptic proteins in a range of syndromes, many of which feature psychosis (reviewed in [30]). These studies have drawn strength from a robust methodological paradigm based on cell-based assays, which express receptors in their natural conformational state at the cell membrane of live eukaryotic cells. Although the phenotype, pathology, and treatment of NMDAR encephalitis [66] have been best characterized (see below), a number of specific autoantibody-receptor associations have been identified recently. Crucially, one of the classic and often early features of NMDAR encephalitis is psychosis. 

Antibody reactivity against neuronal cell surface proteins is an established neurological paradigm that is best illustrated by the pathogenicity of autoantibodies against acetylcholine receptor in myasthenia gravis [67]. The demonstration that there may be a subgroup of patients with psychosis whose disease is antibody-mediated is an intriguing development, as it establishes a link between an accepted model of neuroimmunological disease and the presumed role of neurotransmission defects in the evolution of psychotic disease. Recent reports demonstrating the pathogenic effects of antibody binding to receptors and the clinical efficacy of plasmapheresis and immunotherapy as treatment modalities in NMDAR encephalitis have provided further incentive for the continued exploration of autoantibodies in psychosis [66, 68].

 Antibodies to NMDAR [16–18] and the voltage-gated potassium channel complex (VGKC) [16] have been described in patients with schizophrenia. Although NMDAR encephalitis and schizophrenia are clinically distinct syndromes, the link between psychosis and antibody-associated disease has been reinforced by the detection of anti-NMDAR antibodies in patients with psychosis only. Further studies are needed to assess the prevalence and role of antibodies in disease pathogenesis and the extent of clinical improvement upon immunotherapy.

### 4.1. Early Autoantibody Findings

In the context of schizophrenia, serum antibodies have been reported as early as 1937 [69], but attempts to detect antibodies against diffuse CNS targets have yielded inconsistent and difficult-to-reproduce results [20]. Likewise, the screening of patients for systemic autoantibodies known to be present in other conditions has not validated any particular hypothesis, and measures of total immunoglobulin levels have provided mixed results that are difficult to disentangle from background physiological variation [20]. Investigation of autoantibodies against neurotransmitter receptors in patients with a range of psychiatric disease has resulted in greater specificity [70]. Tanaka and colleagues reported antibodies to muscarinic cholinergic receptor 1, *μ*-opioid receptor, 5-hydroxytryptamine 1A receptor (serotonin receptor), and dopamine-2 receptor in 122 psychiatric patients, including 44 patients with schizophrenic disorders [70]. This study was performed using a radioligand assay requiring the use of isolated recombinant proteins. Although more recent assays have used different methods to express putative autoantigens, the authors' findings foreshadowed current developments in the field of antibody-associated psychiatric disease. Autoantibodies against the dopamine-1 and dopamine-2 receptors have also been detected by Western blot and ELISA in Sydenham chorea and other streptococcal-associated neuropsychiatric disorders [71]. Our group has previously reported immunoglobulin G (IgG) binding to the surface of neuron-like and dopaminergic cells in Sydenham chorea, measured by a cell-based assay utilizing flow cytometry [72]. 

### 4.2. Methods for Antibody Detection

As we have already discussed, a key challenge in establishing immune-mediated mechanisms of neuropsychiatric disease is the demonstration that the immune mechanisms in question are specific. Specificity has been achieved in the context of surface antibody-mediated hypotheses by application of the cell-based assay paradigm [31, 32, 73]. Broadly, a cell line with low or no expression of the antigen of interest is cultured. Cells are then transfected or transduced to express the antigen of interest. Export of the antigenic protein to the cell membrane is confirmed, and then these antigen-expressing cells are used to determine the immunoreactivity of patient samples. Both fluorescence microscopy and flow cytometry have been applied to detect various autoantigens of the CNS, yielding reproducible results [74–79]. The autoantigens studied have included proteins expressed on non-neuronal cells in nonpsychotic diseases, such as myelin oligodendrocyte glycoprotein (MOG) and aquaporin-4 in inflammatory demyelinating CNS disease [75, 77, 79]. The expression of the antigen of interest on the surface of the cell membrane is critical, as it allows determination of specific immunoreactivity to the likely target epitope on the extracellular domain. 

A range of methods other than cell-based assay have been employed to screen for and validate the detection of autoantibodies in neuropsychiatric disease. Immunoblotting using a range of protein extracts has been used to screen for possible antigens but with controversial results. Immunohistochemistry on brain sections from animal models has demonstrated that autoantibodies bind to CNS cells, sometimes in a region-specific manner [80]. Immunocytochemistry on cultured neurons has also been utilised to visualise antibody binding on the cell surface [34, 74, 78]. Ideally, assays for the detection of autoantibodies to cell surface structures should not use denatured, linearized, or intracellular epitopes. 

Although studies examining the frequency of autoantibodies in psychosis have used only cell-based assay for antibody detection, validation of detected antibodies by other methodologies is advisable [32]. In previous studies, antibodies that are detected by cell-based assay have also been visualised by immunocytochemistry and found to bind to brain tissue by immunohistochemistry. An absence of binding of antibody-positive serum in knockout animal models can support the specificity of the proposed antigen [34, 81]. Robust detection paradigms can then lay the foundation for further studies to explore the pathogenic potential of autoantibodies, as has been well documented in the case of NMDAR encephalitis [82, 83].

### 4.3. Autoantibodies Associated with Cell Surface Antigens: A Spectrum of Neuropsychiatric Disease

A new class of neuropsychiatric syndromes has emerged over the past five years. These syndromes share some clinical features and are thought to be mediated by the binding of autoantibodies to antigens at the cell membrane of neuronal cells [84, 85]. There are many excellent reviews of this field [30–32], and we will here only sketch key clinical features and experimental findings relevant to the problem of psychosis. 

In the context of these new neuropsychiatric syndromes, a range of antigens involved in neurotransmission have been identified in autoimmune forms of encephalitides, or “inflammation of the brain parenchyma”. These include the N-terminus extracellular domain of the NR1 subunit of NMDAR, the *α*-amino-3-hydroxy-5-methyl-4-isoxazolepropionic acid receptor (AMPAR), the *γ*-aminobutyric acid type B receptor (GABA_B_R), the metabotropic glutamate receptor 5 (mGluR5), the glycine receptor (GlyR), and various components of the voltage-gated potassium channel complex (VGKC-complex), specifically, leucine-rich glioma inactivated 1 (LGI1), contactin-associated protein-like 2 (CASPR2) and contactin-2 [30–33, 81, 86–89]. Antibody-associated syndromes are a frequent cause of encephalitis, with the incidence of NMDAR encephalitis exceeding most viral causes [90, 91]. The existence and pathogenicity of autoantibodies against CNS antigens were originally established in the context of paraneoplastic disease [74, 92]. Indeed, tumours have been reported with varying frequencies in recently described antibody-associated encephalitides, and tumour removal can be partially therapeutic in those cases where tumours are identified [31]. However, many patients who present with these syndromes do not have a tumour causing paraneoplastic phenomena, and tumour identification is not required for diagnosis [30–32]. 

Antibody-associated neuropsychiatric disorders present with a range of clinical features, some of which are more commonly associated with the presence of certain autoantibodies [31]. Many clinical features are shared between phenotypes. Examples of neurological symptoms include seizures, aphasia, movement disorders, dementia, peripheral nerve hyperexcitability, and rigidity. Psychosis has been described in encephalitis associated with antibodies to NMDAR, CASPR2, AMPAR, and GABA_B_R [30]. The psychiatric features of these syndromes can dominate the initial presentation to the extent that a patient's first contact with health services may be psychiatric. Indeed, it has been proposed that some diagnoses of schizophrenia actually represent patients with undetected NMDAR encephalitis [93]. Although the focus of this review is psychosis, the other psychiatric symptoms present in antibody-associated disorders may be more common and more prominent [30–32]. So far, it has been difficult to clarify the relationship between psychiatric symptoms and specific antibodies (Table 1). Further studies would improve the specificity of proposed associations between psychiatric phenotype and detected autoantibodies.

NMDAR encephalitis is associated with IgG autoantibodies against the NR1 subunit of the NMDAR. These autoantibodies are detectable in both serum and CSF though detection in CSF is thought to be more sensitive [94]. Intrathecal synthesis of antibodies likely plays a role in this syndrome, as is suggested by the infiltration of plasma cells into the CNS of affected patients [95]. Clinical improvement has been observed with the use of steroids, intravenous immunoglobulin, and plasmapheresis as first-line therapy, with rituximab and cyclophosphamide as second-line if necessary [90]. Furthermore, treatment directed only at reducing antibody concentration in serum can be ineffective, and for this reason immune therapies that depress or alter lymphocyte function are probably required in all but the mildest cases. It has also been suggested that intrathecally delivered immune modulators such as methotrexate may be useful as second- or third-line therapy although the side effect profile may be problematic [96]. The role of other monoclonal antibodies, such as alemtuzumab, is unclear. Limited evidence is available to guide treatment in the event that first- and second-line therapies fail [94]. 

The antibodies associated with these syndromes likely have some pathogenic role, as is suggested by the clinical improvement that typically follows immunotherapy [97] and the relationship between serum antibody titers and clinical status [84]. Evidence for pathogenicity has been provided in the case of antibodies against NMDAR [82] but is otherwise scarce for the other autoantibodies. Autoantibody binding to the NMDAR results in a selective decrease in NMDAR surface density [83]. The mechanism involved is likely antibody-mediated capping and internalisation of the receptor. Whilst this mechanism seems to be reversible, and a substantial proportion of patients with NMDAR encephalitis completely recover, there is a subgroup of patients who are left with permanent disability, and in whom other irreversible pathogenic mechanisms may be more prominent [97]. 

Our group has recently published data regarding antibodies to the dopamine-2 receptor (D2R) in patients with autoimmune movement disorders [34]. Using a flow cytometry cell-based assay and immunocytochemistry, antibodies to D2R were detected in 12 of 17 paediatric patients with basal ganglia encephalitis, a neuropsychiatric disorder characterised by parkinsonism, dystonia, chorea, emotional lability, attention deficit, and psychosis. These antibodies were of the IgG class. No anti-D2R antibodies could be found in 67 children with other inflammatory or genetic brain conditions, nor in 22 patients with paediatric autoimmune neuropsychiatric disorders associated with streptococcal infection. However, elevated D2R antibody was detected in 10 of 30 patients with Sydenham chorea and 4 of 44 patients with Tourette's syndrome. The detection of antibodies to the D2R receptor in these cohorts is consistent with the well-substantiated role of dopaminergic pathways in movement and psychiatric disorders [7, 98, 99]. 

### 4.4. Detection of Autoantibodies by Cell-Based Assay in Purely Psychiatric Presentations of Psychosis

Cell-based assays have been employed as a detection technique in patients whose presentations feature psychosis and are purely psychiatric. The first reported use of cell-based assay to identify antibody-positive schizophrenic patients was described by Zandi and colleagues [16]. In a cohort of 46 patients presenting to an early intervention for psychosis service, three patients were identified as positive for NMDAR antibodies and one for VGKC-complex antibodies. Cells were cotransfected with multiple NMDAR subunits, that is, NR1 and NR2B, so the specificity of binding is unclear. The authors noted that they were unable to detect NMDAR antibodies in chronic schizophrenia controls, suggesting that antibody-associated mechanisms of disease may be a transient phenomenon in schizophrenia. Plasmapheresis and oral prednisolone were an effective treatment for one of the NMDAR antibody positive patients described in this study. 

Anti-NMDAR antibodies were also reported by Tsutsui and colleagues [17] in patients with schizophrenia, schizoaffective disorders, and narcolepsy with severe psychosis. Four of 51 patients with schizophrenia and schizoaffective disorders were antibody-positive, and three of five patients with narcolepsy with severe psychosis. Of the latter group, one antibody-positive patient suffered from comorbid Parkinsonism, whilst the other two had no symptoms of neurodegenerative disease. It is noteworthy that three of the patients identified in this cohort as having psychiatric disease also experienced seizures. Furthermore, two of the three patients with seizures were antibody-positive, and two antibody-positive patients had ovarian tumours. Both seizures and ovarian tumours have been described extensively in antibody-associated neuropsychiatric disorders (see Section 4.3 above), and their presence warrants consideration of an autoimmune aetiology.

The largest cohort of schizophrenic patients assessed for the presence of autoantibodies in serum, and CSF, was described by Steiner and colleagues [18]. They compared 121 patients with acute presentations of schizophrenia to 70 patients with major depression, 38 patients with borderline personality disorder, and 230 healthy matched controls. The presence of autoantibodies to NMDAR and AMPAR was assessed by cell-based assay after transfection of NR1 only, or NR1/NR2B subunits of the NMDAR. This study also assessed different classes of antibody, with samples assessed for IgG, IgA, and IgM positivity. 12 of 121 (9.9%) schizophrenic patients were found to be antibody positive in serum, but two were retrospectively deemed to have been misdiagnosed cases of NMDAR encephalitis. This contrasts with substantially fewer antibody-positive samples in the borderline personality disorder (0/38), major depression (2/70), and healthy control (1/230) groups. Although autoantibodies were detected in a number of cases in serum, only two patients had antibodies in CSF, and both patients were retrospectively diagnosed with NMDAR encephalitis. The seropositive cases from the schizophrenia group exhibited antibodies from all three of the immunoglobulin classes (IgG, IgA, and IgM) that were tested. Although most antibodies bound to cells transfected with the NR1 subunit construct, two schizophrenia patients had serum that was immunoreactive against the NR1/NR2B construct, and not the NR1 subunit alone. These findings reinforce the hypothesis that the spectrum of autoantibodies produced in patients with schizophrenia alone is likely different to that to be found in NMDAR encephalitis cohorts; the latter being characterized by immunoreactivity against NR1 alone and autoantibodies predominantly of the IgG1 subtype [31]. Indeed, Masdeu and colleagues were unable to detect IgG antibodies against NR1 in 80 patients diagnosed with schizophrenia [100], leading to the argument that testing for IgG NR1 antibodies in schizophrenia is not indicated [94]. Rhoads and colleagues also reported that none of seven patients with chronic antipsychotic-treated schizophrenia had detectable antibodies to NMDAR [101]. At this stage, it is likely that IgG antibodies against the NR1 subunit of the NMDAR are specific to NMDAR encephalitis rather than schizophrenia, and IgG NR1 antibodies have yet to be found in patients presenting with psychosis alone. Furthermore, the pathogenic potential of the novel antibodies identified by Steiner and colleagues has not yet been explored and warrants further investigation. These novel antibodies are summarized in Table 2. 

The detection of autoantibodies in some schizophrenic patients [16–18] supports an association between autoantibodies against neuronal receptors in a subgroup of patients with psychosis and purely psychiatric disease. Such a subgroup is likely associated with risk factors that have yet to be identified and a phenotype that has yet to be described. Although the cohort compositions and antigen and immunoglobulin subclass vary between published studies (Table 2), they share the direct examination of the frequency of autoantibodies in primary psychotic disease and their use of a similar detection method. Further studies should inform about the time point in the disease process when antibodies likely appear, the pathogenicity of these autoantibodies, and whether or not a range of antibodies lead to a similar clinical phenotype.

Protocols for assessment of patients presenting with the first episode of a psychotic disorder should always include medical screening, to exclude treatable organic causes. This is particularly important in those who present in an acutely confused state and are possibly delirious from an encephalopathic process as opposed to a functional psychosis. Some authors have reported elevated NMDAR antibodies in this group, 3/46 in one study [16], and 12/121 in another [18]. Steiner and colleagues have even suggested that 2/121 patients had NMDAR encephalitis that was misdiagnosed as schizophrenia [18].

Corroboration of experimental findings with the clinical picture is crucial. The possibility of a subgroup of antibody-positive patients with functional psychosis highlights the importance of considering the complete clinical picture, including established investigations for organic disease, in order to differentiate between established antibody-associated neuropsychiatric disease syndromes and purely psychiatric pathology. The effectiveness of immune therapy in antibody-positive patients should also be assessed, so as to relate the proposed model to clinical reality and enable more effective treatment. 

## 5. Conclusions 

Immunological disturbance in psychotic disease is complex. Data about a range of immunological phenomena has only recently started to coalesce into discernible trends and move towards providing information that is useful in a clinical setting. The application of cell-based assay as an antibody detection system has provided clarity to this aspect of neuroimmunological investigation. Although limited, the success of immunotherapy in treating cases of antibody-associated encephalitis and one case of antibody-positive schizophrenia provides some optimism that a new treatment paradigm may be developed based on immune modulation in a subgroup of susceptible patients. Further studies assessing the prevalence and significance of autoantibodies and the effectiveness of immune therapy could potentially influence our understanding and clinical management of the diverse spectrum of psychotic disease. 

## Figures and Tables

**Table 1:** Psychiatric symptoms associated with antibodies to cell surface autoantigens.

Psychiatric symptom	Reported antigens [30–35]
Psychosis	NMDAR, D2R, LGI1, CASPR2, AMPAR, GABA_B_R, mGluR5
Mania	NMDAR
Agitation	NMDAR, D2R, LGI1, CASPR2, AMPAR, GABA_B_R, mGluR5
Emotional lability	NMDAR, D2R, mGluR5
Anxiety	NMDAR, D2R, LGI1, CASPR2, mGluR5
Aggression	NMDAR, D2R
Compulsive behaviour	NMDAR, D2R
Memory impairment, amnesia	NMDAR, LGI1, CASPR2, AMPAR, GABA_B_R, mGluR5
Personality change	NMDAR, D2R, LGI1, CASPR2
Confusion	NMDAR, D2R, LGI1, CASPR2, mGluR5

D2R: dopamine-2 receptor; NMDAR: N-methyl-D-aspartate receptor; LGI1: leucine-rich glioma inactivated 1; CASPR2: contactin-associated protein-like 2; AMPAR: *α*-amino-3-hydroxy-5-methyl-4-isoxazolepropionic acid receptor; GABA_B_R: *γ*-aminobutyric acid type B receptor; mGluR5: metabotropic glutamate receptor 5.

**Table 2:** Frequency of antibody detection in patients with psychosis.

Cohort	Autoantibodies detected			Paper
	Antigen	Ig class	Frequency	
First episode psychosis (*n* = 46)	NMDAR	IgG	3/46 (6.5%)	Zandi and colleagues (2011) [16]
	VGKC	IgG	1/46 (2.2%)	

NMDAR encephalitis (*n* = 5), general psychiatric disease (*n* = 5), schizophrenia and schizoaffective disorders (*n* = 51)	NMDAR	IgG	3/5 (60%) (NMDAR encephalitis) 3/5 (60%) (general psychiatric disease) 4/51 (7.8%) (SZ and schizoaffective disorders)	Tsutsui and colleagues (2012) [17]

Schizophrenia, acutely ill, both first episode and chronic (*n* = 121); major depression, acutely ill (*n* = 70); borderline personality disorder, acutely ill (*n* = 38); matched healthy controls (*n* = 230)		IgG	4/121 (3.3%) (SZ) 0/70 (MD) 0/38 (BLPD) 0/230 (healthy controls)	
	NMDAR	IgA	6/121 (5%) (SZ) 2/70 (2.9%) (MD) 0/38 (BLPD) 0/230 (healthy controls)	Steiner and colleagues (2013) [18]
		IgM	4/121 (3.3%) (SZ) 0/70 (MD) 0/38 (BLPD) 1/230 (0.4%) (healthy controls)	

NMDAR: N-methyl-D-aspartate receptor; VGKC: voltage gated potassium channel; Ig: immunoglobulin, SZ: schizophrenia; MD: major depression; BLPD: borderline personality disorder.

## References

[1] First MB, Frances A, Pincus HA (2004). Schizophrenia and other psychotic disorders. *DSM-IV-TR Guidebook*.

[2] van Os J, Kapur S (2009). Schizophrenia. *The Lancet*.

[3] Löthgren M (2004). Economic evidence in psychotic disorders: a review. *European Journal of Health Economics*.

[4] Rössler W, Joachim Salize HH, van Os J, Riecher-Rössler A (2005). Size of burden of schizophrenia and psychotic disorders. *European Neuropsychopharmacology*.

[5] Rapoport JL, Addington AM, Frangou S, Psych MRC (2005). The neurodevelopmental model of schizophrenia: update 2005. *Molecular Psychiatry*.

[6] Pantelis C, Yücel M, Bora E (2009). Neurobiological markers of illness onset in psychosis and schizophrenia: the search for a moving target. *Neuropsychology Review*.

[7] Beaulieu J-M, Gainetdinov RR (2011). The physiology, signaling, and pharmacology of dopamine receptors. *Pharmacological Reviews*.

[8] Seeman P (1987). Dopamine receptors and the dopamine hypothesis of schizophrenia. *Synapse*.

[9] López-Muñoz F, Álamo C (2011). Neurobiological background for the development of new drugs in schizophrenia. *Clinical Neuropharmacology*.

[10] Friston KJ (1999). Schizophrenia and the disconnection hypothesis. *Acta Psychiatrica Scandinavica, Supplement*.

[11] Kristiansen LV, Huerta I, Beneyto M, Meador-Woodruff JH (2007). NMDA receptors and schizophrenia. *Current Opinion in Pharmacology*.

[12] Coyle JT (2006). Glutamate and schizophrenia: beyond the dopamine hypothesis. *Cellular and Molecular Neurobiology*.

[13] Strous RD, Shoenfeld Y (2006). Schizophrenia, autoimmunity and immune system dysregulation: a comprehensive model updated and revisited. *Journal of Autoimmunity*.

[14] Jones AL, Mowry BJ, Pender MP, Greer JM (2005). Immune dysregulation and self-reactivity in schizophrenia: do some cases of schizophrenia have an autoimmune basis?. *Immunology and Cell Biology*.

[15] Fudenberg HH, Whitten HD, Merler E, Farmati O (1983). Is schizophrenia an immunologic receptor disorder?. *Medical Hypotheses*.

[16] Zandi MS, Irani SR, Lang B (2011). Disease-relevant autoantibodies in first episode schizophrenia. *Journal of Neurology*.

[17] Tsutsui K, Kanbayashi T, Tanaka K (2012). Anti-NMDA-receptor antibody detected in encephalitis, schizophrenia, and narcolepsy with psychotic features. *BMC Psychiatry*.

[18] Steiner J, Walter M, Glanz W (2013). Increased prevalence of diverse N-methyl-D-aspartate glutamate receptor antibodies in patients with an initial diagnosis of schizophrenia: specific relevance of IgG NR1a antibodies for distinction from N-methyl-D-aspartate glutamate receptor encephalitis. *JAMA Psychiatry*.

[19] Kinney DK, Hintz K, Shearer EM (2010). A unifying hypothesis of schizophrenia: abnormal immune system development may help explain roles of prenatal hazards, post-pubertal onset, stress, genes, climate, infections, and brain dysfunction. *Medical Hypotheses*.

[20] Rothermundt M, Arolt V, Bayer TA (2001). Review of immunological and immunopathological findings in schizophrenia. *Brain, Behavior, and Immunity*.

[21] Ganguli R, Brar JS, Rabin BS (1994). Immune abnormalities in schizophrenia: evidence for the autoimmune hypothesis. *Harvard Review of Psychiatry*.

[22] Menninger KA (1928). The schizophrenic syndrome as a product of acute infectious disease. *Archives of Neurology and Psychiatry*.

[23] Kirch DG (1993). Infection and autoimmunity as etiologic factors in schizophrenia: a review and reappraisal. *Schizophrenia Bulletin*.

[24] Chen S-J, Chao Y-L, Chen C-Y (2012). Prevalence of autoimmune diseases in in-patients with schizophrenia: nationwide population-based study. *British Journal of Psychiatry*.

[25] Eaton WW, Pedersen MG, Nielsen PR, Mortensen PB (2011). Autoimmune diseases, bipolar disorder, and non-affective psychosis. *Bipolar Disorders*.

[26] Eaton WW, Byrne M, Ewald H (2006). Association of schizophrenia and autoimmune diseases: linkage of Danish national registers. *American Journal of Psychiatry*.

[27] Busse S, Busse M, Schiltz K (2012). Different distribution patterns of lymphocytes and microglia in the hippocampus of patients with residual versus paranoid schizophrenia: further evidence for disease course-related immune alterations?. *Brain, Behavior, and Immunity*.

[28] Dickerson F, Stallings C, Origoni A (2013). C-reactive protein is elevated in schizophrenia. *Schizophrenia Research*.

[29] Fillman SG, Cloonan N, Catts VS (2013). Increased inflammatory markers identified in the dorsolateral prefrontal cortex of individuals with schizophrenia. *Molecular Psychiatry*.

[30] Zuliani L, Graus F, Giometto B, Bien C, Vincent A (2012). Central nervous system neuronal surface antibody associated syndromes: review and guidelines for recognition. *Journal of Neurology, Neurosurgery and Psychiatry*.

[31] Vincent A, Bien CG, Irani SR, Waters P (2011). Autoantibodies associated with diseases of the CNS: new developments and future challenges. *The Lancet Neurology*.

[32] Lancaster E, Dalmau J (2012). Neuronal autoantigens—pathogenesis, associated disorders and antibody testing. *Nature Reviews*.

[33] Lancaster E, Martinez-Hernandez E, Titulaer MJ (2011). Antibodies to metabotropic glutamate receptor 5 in the Ophelia syndrome. *Neurology*.

[34] Dale R, Merheb V, Pillai S Antibodies to surface dopamine-2 receptor in autoimmune movement and psychiatric disorders. *Brain*.

[35] Irani SR, Pettingill P, Kleopa KA (2012). Morvan syndrome: clinical and serological observations in 29 cases. *Annals of Neurology*.

[36] Gaughran F (2002). Immunity and schizophrenia: autoimmunity, cytokines, and immune responses. *International Review of Neurobiology*.

[37] Hickey WF (2001). Basic principles of immunological surveillance of the normal central nervous system. *GLIA*.

[38] Ousman SS, Kubes P (2012). Immune surveillance in the Central Nervous System. *Nature Neuroscience*.

[39] Bechter K, Reiber H, Herzog S, Fuchs D, Tumani H, Maxeiner HG (2010). Cerebrospinal fluid analysis in affective and schizophrenic spectrum disorders: identification of subgroups with immune responses and blood-CSF barrier dysfunction. *Journal of Psychiatric Research*.

[40] Daneman R (2012). The blood-brain barrier in health and disease. *Annals of Neurology*.

[41] Brilot F (2010). Inflammation and autoimmunity: a nervous system perspective. *Inflammatory and Autoimmune Disorders of the Nervous System in Children*.

[42] Ripke S, Sanders AR, Kendler KS (2011). Genome-wide association study identifies five new schizophrenia loci. *Nature Genetics*.

[43] Liu J, Li J, Li T (2011). CTLA-4 confers a risk of recurrent schizophrenia, major depressive disorder and bipolar disorder in the Chinese Han population. *Brain, Behavior, and Immunity*.

[44] Michel M, Schmidt MJ, Mirnics K (2012). Immune system gene dysregulation in autism and schizophrenia. *Developmental Neurobiology*.

[45] Gilvarry CM, Sham PC, Jones PB (1996). Family history of autoimmune diseases in psychosis. *Schizophrenia Research*.

[46] Martínez-Gras I, García-Sánchez F, Guaza C (2012). Altered immune function in unaffected first-degree biological relatives of schizophrenia patients. *Psychiatry Research*.

[47] Potvin S, Stip E, Sepehry AA, Gendron A, Bah R, Kouassi E (2008). Inflammatory cytokine alterations in schizophrenia: a systematic quantitative review. *Biological Psychiatry*.

[48] Fineberg AM, Ellman LM (2013). Inflammatory cytokines and neurological and neurocognitive alterations in the course of schizophrenia. *Biological Psychiatry*.

[49] Miller BJ, Buckley P, Seabolt W, Mellor A, Kirkpatrick B (2011). Meta-analysis of cytokine alterations in schizophrenia: clinical status and antipsychotic effects. *Biological Psychiatry*.

[50] Mansur RB, Zugman A, Asevedo EDM (2012). Cytokines in schizophrenia: possible role of anti-inflammatory medications in clinical and preclinical stages. *Psychiatry and Clinical Neurosciences*.

[51] Smith RS, Maes M (1995). The Macrophage-T-lymphocyte theory of schizophrenia: additional evidence. *Medical Hypotheses*.

[52] García-Bueno B, Bioque M, Mac-Dowell KS Pro-/anti-inflammatory dysregulation in patients with first episode of psychosis: toward an integrative inflammatory hypothesis of schizophrenia.

[53] Steiner J, Mawrin C, Ziegeler A (2006). Distribution of HLA-DR-positive microglia in schizophrenia reflects impaired cerebral lateralization. *Acta Neuropathologica*.

[54] Frick LR, Williams K, Pittenger C (2013). Microglial dysregulation in psychiatric disease. *Clinical and Developmental Immunology*.

[55] Meyer U, Schwarz MJ, Müller N (2011). Inflammatory processes in schizophrenia: a promising neuroimmunological target for the treatment of negative/cognitive symptoms and beyond. *Pharmacology and Therapeutics*.

[56] Sommer IE, De Witte L, Begemann M, Kahn RS (2012). Nonsteroidal anti-inflammatory drugs in schizophrenia: ready for practice or a good start? A meta-analysis. *Journal of Clinical Psychiatry*.

[57] Müller N, Empl M, Riedel M, Schwarz M, Ackenheil M (1997). Neuroleptic treatment increases soluble IL-2 receptors and decreases soluble IL-6 receptors in schizophrenia. *European Archives of Psychiatry and Clinical Neuroscience*.

[58] Pollmächer T, Haack M, Schuld A, Kraus T, Hinze-Selch D (2000). Effects of antipsychotic drugs on cytokine networks. *Journal of Psychiatric Research*.

[59] Drzyzga Ł, Obuchowicz E, Marcinowska A, Herman ZS (2006). Cytokines in schizophrenia and the effects of antipsychotic drugs. *Brain, Behavior, and Immunity*.

[60] Ohaeri JU, Akanji AO (2011). Metabolic syndrome in severe mental disorders. *Metabolic Syndrome and Related Disorders*.

[61] Saha S, Chant D, McGrath J (2007). A systematic review of mortality in schizophrenia: is the differential mortality gap worsening over time?. *Archives of General Psychiatry*.

[62] Miller BJ, Culpepper N, Rapaport MH, Buckley P (2013). Prenatal inflammation and neurodevelopment in schizophrenia: a review of human studies. *Progress in Neuro-Psychopharmacology and Biological Psychiatry*.

[63] Grose C, Itani O, Weiner CP (1989). Prenatal diagnosis of fetal infection: advances from amniocentesis to cordocentesis—congenital toxoplasmosis, rubella, cytomegalovirus, varicella virus, parvovirus and human immunodeficiency virus. *Pediatric Infectious Disease Journal*.

[64] Boksa P (2010). Effects of prenatal infection on brain development and behavior: a review of findings from animal models. *Brain, Behavior, and Immunity*.

[65] Khandaker GM, Zimbron J, Lewis G, Jones PB (2013). Prenatal maternal infection, neurodevelopment and adult schizophrenia: a systematic review of population-based studies. *Psychological Medicine*.

[66] Dalmau J, Gleichman AJ, Hughes EG (2008). Anti-NMDA-receptor encephalitis: case series and analysis of the effects of antibodies. *The Lancet Neurology*.

[67] Vincent A, Palace J, Hilton-Jones D (2001). Myasthenia gravis. *The Lancet*.

[68] Dalmau J, Lancaster E, Martinez-Hernandez E, Rosenfeld MR, Balice-Gordon R (2011). Clinical experience and laboratory investigations in patients with anti-NMDAR encephalitis. *The Lancet Neurology*.

[69] Lehmann-Facius H (1937). Über die liquordiagnose der schizophrenien. *Klinische Wochenschrift*.

[70] Tanaka S, Matsunaga H, Kimura M (2003). Autoantibodies against four kinds of neurotransmitter receptors in psychiatric disorders. *Journal of Neuroimmunology*.

[71] Brimberg L, Benhar I, Mascaro-Blanco A (2012). Behavioral, pharmacological, and immunological abnormalities after streptococcal exposure: a novel rat model of sydenham chorea and related neuropsychiatric disorders. *Neuropsychopharmacology*.

[72] Brilot F, Merheb V, Ding A, Murphy T, Dale RC (2011). Antibody binding to neuronal surface in Sydenham chorea, but not in PANDAS or Tourette syndrome. *Neurology*.

[73] Kleopa KA, Elman LB, Lang B, Vincent A, Scherer SS (2006). Neuromyotonia and limbic encephalitis sera target mature *Shaker*-type K^+^ channels: subunit specificity correlates with clinical manifestations. *Brain*.

[74] Dalmau J, Tüzün E, Wu H (2007). Paraneoplastic anti-N-methyl-D-aspartate receptor encephalitis associated with ovarian teratoma. *Annals of Neurology*.

[75] McLaughlin KA, Chitnis T, Newcombe J (2009). Age-dependent B cell autoimmunity to a myelin surface antigen in pediatric multiple sclerosis. *Journal of Immunology*.

[76] Dale RC, Irani SR, Brilot F (2009). N-methyl-D-aspartate receptor antibodies in pediatric dyskinetic encephalitis lethargica. *Annals of Neurology*.

[77] Brilot F, Dale RC, Selter RC (2009). Antibodies to native myelin oligodendrocyte glycoprotein in children with inflammatory demyelinating central nervous system disease. *Annals of Neurology*.

[78] Irani SR, Alexander S, Waters P (2010). Antibodies to Kv1 potassium channel-complex proteins leucine-rich, glioma inactivated 1 protein and contactin-associated protein-2 in limbic encephalitis, Morvan’s syndrome and acquired neuromyotonia. *Brain*.

[79] Waters PJ, McKeon A, Leite MI (2012). Serologic diagnosis of NMO: a multicenter comparison of aquaporin-4-IgG assays. *Neurology*.

[80] Ances BM, Vitaliani R, Taylor RA (2005). Treatment-responsive limbic encephalitis identified by neuropil antibodies: MRI and PET correlates. *Brain*.

[81] Lai MZ, Huijbers MGM, Lancaster E (2010). Investigation of LGI1 as the antigen in limbic encephalitis previously attributed to potassium channels: a case series. *Lancet Neurology*.

[82] Mikasova L, De Rossi P, Bouchet D (2012). Disrupted surface cross-talk between NMDA and Ephrin-B2 receptors in anti-NMDA encephalitis. *Brain*.

[83] Hughes EG, Peng X, Gleichman AJ (2010). Cellular and synaptic mechanisms of anti-NMDA receptor encephalitis. *The Journal of Neuroscience*.

[84] Graus F, Saiz A, Dalmau J (2010). Antibodies and neuronal autoimmune disorders of the CNS. *Journal of Neurology*.

[85] Lancaster E, Martinez-Hernandez E, Dalmau J (2011). Encephalitis and antibodies to synaptic and neuronal cell surface proteins. *Neurology*.

[86] Gleichman AJ, Spruce LA, Dalmau J (2012). Anti-NMDA receptor encephalitis antibody binding is dependent on amino acid identity of a small region within the GluN1 amino terminal domain. *The Journal of Neuroscience*.

[87] Hutchinson M, Waters P, McHugh J (2008). Progressive encephalomyelitis, rigidity, and myoclonus: a novel glycine receptor antibody. *Neurology*.

[88] Lai MZ, Hughes EG, Peng XY (2009). AMPA receptor antibodies in limbic encephalitis alter synaptic receptor location. *Annals of Neurology*.

[89] Lancaster E, Lai M, Peng X (2010). Antibodies to the GABAB receptor in limbic encephalitis with seizures: case series and characterisation of the antigen. *The Lancet Neurology*.

[90] Titulaer MJ, McCracken L, Gabilondo I (2013). Treatment and prognostic factors for long-term outcome in patients with anti-NMDA receptor encephalitis: an observational cohort study. *Lancet Neurology*.

[91] Gable MS, Sheriff H, Dalmau J, Tilley DH, Glaser CA (2012). The frequency of autoimmune N-methyl-D-aspartate receptor encephalitis surpasses that of individual viral etiologies in young individuals enrolled in the california encephalitis project. *Clinical Infectious Diseases*.

[92] Dalmau J, Rosenfeld MR (2008). Paraneoplastic syndromes of the CNS. *The Lancet Neurology*.

[93] Lennox BR, Coles AJ, Vincent A (2012). Antibody-mediated encephalitis: a treatable cause of schizophrenia. *The British Journal of Psychiatry*.

[94] Titulaer MJ, Kayser MS, Dalmau J (2013). Prevalence and treatment of anti-NMDA receptor encephalitis. *Lancet Neurology*.

[95] Martinez-Hernandez E, Horvath J, Shiloh-Malawsky Y, Sangha N, Martinez-Lage M, Dalmau J (2011). Analysis of complement and plasma cells in the brain of patients with anti-NMDAR encephalitis. *Neurology*.

[96] Liba Z, Sebronova V, Komarek V (2013). Prevalence and treatment of anti-NMDA receptor encephalitis. *Lancet Neurology*.

[97] Irani SR, Vincent A (2011). NMDA receptor antibody encephalitis. *Current Neurology and Neuroscience Reports*.

[98] Nikolaus S, Antke C, Müller H-W (2009). In vivo imaging of synaptic function in the central nervous system. I. Movement disorders and dementia. *Behavioural Brain Research*.

[99] Nikolaus S, Antke C, Müller H-W (2009). In vivo imaging of synaptic function in the central nervous system: II. Mental and affective disorders. *Behavioural Brain Research*.

[100] Masdeu JC, Gonzalez-Pinto A, Matute C (2012). Serum IgG antibodies against the NR1 subunit of the NMDA receptor not detected in schizophrenia. *American Journal of Psychiatry*.

[101] Rhoads J, Guirgis H, McKnight C, Duchemin A-M (2011). Lack of anti-NMDA receptor autoantibodies in the serum of subjects with schizophrenia. *Schizophrenia Research*.

